# Activity of the DNA minor groove cross-linking agent SG2000 (SJG-136) against canine tumours

**DOI:** 10.1186/s12917-015-0534-2

**Published:** 2015-08-19

**Authors:** Maria Mellinas-Gomez, Victoria J. Spanswick, Solange R. Paredes-Moscosso, Matthew Robson, R. Barbara Pedley, David E. Thurston, Stephen J. Baines, Anneliese Stell, John A. Hartley

**Affiliations:** CR-UK Drug-DNA Interactions Research Group, UCL Cancer Institute, Paul O’Gorman Building, University College London, 72 Huntley Street, London, WC1E 6BT UK; UCL Cancer Institute, Paul O’Gorman Building, 72 Huntley Street, London, WC1E 6BT UK; The School of Pharmacy, University College London, Brunswick Square, London, WC1E 6BT UK; Royal Veterinary College, Hawkshead Lane, North Mymms, Hatfield, Herts, AL9 7TA UK; Present address: Institute of Pharmaceutical Science, King’s College London, Britannia House, 7 Trinity Street, London, SE1 1DB UK; Present address: Willows Referral Service, Highlands Road, Shirley, Solihull, West Midlands, B90 4NH UK

**Keywords:** Canine cancer, SG2000 (SJG-136), DNA interstrand cross-linking, Anticancer drug, Pyrrolobenzodiazepine dimer, Cancer chemotherapy

## Abstract

**Background:**

Cancer is the leading cause of death in older dogs and its prevalence is increasing. There is clearly a need to develop more effective anti-cancer drugs in dogs. SG2000 (SJG-136) is a sequence selective DNA minor groove cross-linking agent. Based on its *in vitro* potency, the spectrum of *in vivo* and clinical activity against human tumours, and its tolerability in human patients, SG2000 has potential as a novel therapeutic against spontaneously occurring canine malignancies.

**Results:**

*In vitro* cytotoxicity was assessed using SRB and MTT assays, and *in vivo* activity was assessed using canine tumour xenografts. DNA interstrand cross-linking (ICL) was determined using a modification of the single cell gel electrophoresis (comet) assay. Effects on cell cycle distribution were assessed by flow cytometry and measurement of γ-H2AX by immunofluorescence and immunohistochemistry.

SG2000 had a multi-log differential cytotoxic profile against a panel of 12 canine tumour cell lines representing a range of common tumour types in dogs. In the CMeC-1 melanoma cell line, DNA ICLs increased linearly with dose following a 1 h treatment. Peak ICL was achieved within 1 h and no removal was observed over 48 h. A relationship between DNA ICL formation and cytotoxicity was observed across cell lines. The formation of γ-H2AX foci was slow, becoming evident after 4 h and reaching a peak at 24 h.

SG2000 exhibited significant anti-tumour activity against two canine melanoma tumour models *in vivo*. Anti-tumour activity was observed at 0.15 and 0.3 mg/kg given i.v. either once, or weekly x 3. Dose-dependent DNA ICL was observed in tumours (and to a lower level in peripheral blood mononuclear cells) at 2 h and persisted at 24 h. ICL increased following the second and third doses in a repeated dose schedule. At 24 h, dose dependent γ-H2AX foci were more numerous than at 2 h, and greater in tumours than in peripheral blood mononuclear cells. SG2000-induced H2AX phosphorylation measured by immunohistochemistry showed good correspondence, but less sensitivity, than measurement of foci.

**Conclusions:**

SG2000 displayed potent activity *in vitro* against canine cancer cell lines as a result of the formation and persistence of DNA ICLs. SG2000 also had significant *in vivo* antitumour activity against canine melanoma xenografts, and the comet and γ-H2AX foci methods were relevant pharmacodynamic assays. The clinical testing of SG2000 against spontaneous canine cancer is warranted.

**Electronic supplementary material:**

The online version of this article (doi:10.1186/s12917-015-0534-2) contains supplementary material, which is available to authorized users.

## Background

Cancer is the leading cause of death in older dogs and its prevalence is increasing [1, 2]. The skin and soft tissues are common sites for tumour development with mast cell tumours being the most common malignant skin tumour. Mammary tumours and haematological malignancies are also frequently diagnosed [3]. Current therapies in canine oncology, including chemotherapy, are often extrapolated from those known to be effective in the corresponding human disease. There are currently few veterinary licenced drugs for the treatment of cancer in dogs. Although chemo-sensitive tumours can be treated successfully initially, when conventional treatment fails, few alternative options exist. In addition, some types of tumours are intrinsically drug resistant (e.g. malignant melanoma and many carcinomas). There is clearly a need to develop novel effective agents with an acceptable toxicity profile targeted towards the most common canine malignancies.

SG2000 (SJG-136, NSC694501) is a rationally designed pyrrolobenzodiazepine dimer which interacts sequence selectively in the minor groove of DNA [4, 5]. It spans six base pairs with a preference for 5’-purine-GATC-pyrimidine sequences, binding covalently to the N2-positions of guanine on opposite strands of the DNA molecule to form highly cytotoxic DNA interstrand cross-links (ICLs) spanning four base pairs [5–7]. It can also form intrastrand cross-links and monoadducts [8] and the ratio of the three types of adducts can depend on sequence [9]. SG2000 was found to have potent multi-log differential activity in the National Cancer Institute anticancer drug screen with a mean GI_50_ (dose of SG2000 which gives 50 % inhibition of cell growth) of 7.4 nM (range 0.14 to 320 nM) [7]. The NCI standard COMPARE and molecular target analysis of the screening data suggested that the drug had a distinct mechanism of action since, although it had some similarity to other DNA binding agents, it did not fit within the clusters of known agents [7]. Based on these impressive *in vitro* data and significant activity in the NCI standard hollow fiber assay [7], SG2000 was tested extensively *in vivo* against human tumour xenografts [7, 10]. In ten tumour models tested by the NCI (including melanoma, breast, colon, lung and ovarian carcinomas, brain tumours and leukemia), SG2000 was active against small (150 mg) and large (250-400 mg) xenografts with tumour mass reductions in all ten models [10]. Pharmacokinetic studies in rats [11] and dogs [12] also reported peak plasma concentrations following a single dose of SG2000 within the range of concentrations associated with *in vitro* DNA ICL and anti-proliferative activity.

Based on the large body of data showing activity and tolerability in preclinical studies, SG2000 entered clinical Phase I testing in humans against both solid tumours and haematological malignancies. Results from three of these studies using different dosing schedules have been reported [13–15] and the agent has progressed to human Phase II clinical trials. Dose limiting toxicities included edema, dyspnea, fatigue and delayed liver toxicity. No significant myelotoxicity was observed. The *in vitro* potency, alongside the tolerability and broad spectrum activity of SG2000 against human tumours *in vivo* (with breast carcinoma, melanoma and haematological malignancies being amongst the most sensitive), suggests that this agent is a promising candidate as a novel cancer therapeutic against spontaneously occurring malignancies in dogs. The current study was therefore undertaken to investigate the activity and cellular pharmacology of SG2000 in canine cancers *in vitro*, to assess the *in vivo* antitumour activity of SG2000 against canine tumour xenografts and to evaluate the potential of the comet and γ-H2AX foci methods as pharmacodynamic assays for use in the further clinical development of the drug.

## Methods

### Canine cell lines

CMeC-1, CMeC-2, KMeC, LMeC melanoma cell lines [16] were provided by Professor Nobuo Sasaki (University of Tokyo); the DEN haemangiosarcoma cell line [17] by Professor Douglas Thamm (Colorado State University); the melanoma 12 cell line [18] by Professor Michael Kent (University of California, Davis). The ARCE mast cell tumour line was provided by Dr Richard Elders (formerly RVC, University of London, now at University of Edinburgh). The canine cell lines C2, DH82, A72, D17, CF33MG, CF35MG and MDCK and the human melanoma cell line LOXIMVI, were obtained from ATCC.

### Cell culture

Cell cultures were maintained in exponential growth with the appropriate supplemented media in 75 mL cell culture flasks, at 37 °C and 5 % CO_2_, in a humidified atmosphere. EMEM (Eagle’s Minimal Essential Medium), DMEM (Dulbecco Modified Eagle Medium) and RPMI (Royal Park Memorial Institute) (PAA Laboratories GmbH, UK) media were supplemented with heat inactivated foetal calf serum (FCS), (Source BioScience, UK), glutamine (Source BioScience, UK) and non-essential amino acids (NEAA) (Source BioScience, UK), as required for the individual cell lines as shown in Additional file 1: Table S1.

### Drug

Pyrrolo[2,1-*c*][1,4]benzodiazepine dimer SG2000 (SJG-136, BN2629, NSC694501) was obtained from Ipsen Ltd, (Slough, UK) in powder form and dissolved in dimethylsulfoxide (DMSO) (Sigma-Aldrich Co, UK) to provide a 10 mM stock solution, stored at -20 °C. Further dilutions of SG2000 were prepared fresh from this stock in cell culture medium to provide the desired concentration.

### Cell growth inhibition assays

*In vitro* growth inhibition of SG2000 was assessed using either the sulphorhodamine B (SRB) assay for adherent cell lines or the methyl-3-(4,5-dimethylthiazol-2-yl)-2,5-diphenyltetrazolium bromide (MTT) assay for suspension cell lines as previously described [7]. SG2000 was used at a concentration range of 0.03 nM to 1000 nM. Cytotoxic effects of SG2000 were measured either after 1 h (followed by 96 h in drug-free medium) or following continuous (96 h) exposure and the GI_50_ dose was determined for each cell line. All chemicals were obtained from Sigma-Aldrich (Poole, UK) unless specified.

### Determination of DNA interstrand cross-link formation and its repair using the single cell gel electrophoresis (comet) assay

The details of the single cell gel electrophoresis (comet) assay used to measure DNA ICL formation and repair are described in detail elsewhere [19]. All procedures were carried out on ice and in subdued lighting. Chemicals were obtained from Sigma-Aldrich Co. (Poole, U.K.) unless otherwise stated. Immediately before analysis cells were diluted to give a final concentration of 2.5 ×10^4^ cells/mL and irradiated (15 Gy) in order to deliver a fixed number of random DNA strand breaks. After embedding cells in 1 % agarose on a pre-coated microscope slide, the cells were lysed for 1 h in lysis buffer (100 mM disodium EDTA, 2.5 M NaCl, 10 mM Tris–HCl pH 10.5) containing 1 % Triton X-100 added immediately before analysis, and then washed every 15 min in distilled water for 1 h. Slides were then incubated in alkali buffer (50 mM NaOH, 1 mM disodium EDTA, pH12.5) for 45 min followed by electrophoresis in the same buffer for 25 min at 18 V (0.6 V/cm), 250 mA. The slides were rinsed in neutralising buffer (0.5 M Tris–HCl, pH 7.5) then saline.

After drying, the slides were stained with propidium iodide (2.5 μg/mL) for 30 min then rinsed in distilled water. Images were visualised using a NIKON inverted microscope with high-pressure mercury light source, 510-560 nm excitation filter and 590 nm barrier filter at ×20 magnification. Images were captured using an on-line CCD camera and analysed using Komet Analysis software 6.0 (Andor Technology, U.K.). For each duplicate slide, 25 cells were analysed. The tail moment for each image was calculated as the product of the percentage DNA in the comet tail and the distance between the means of the head and tail distributions [20]. DNA interstrand cross-linking was expressed as percentage decrease in tail moment compared to irradiated controls calculated by the formula: % decrease in tail moment $=\left[1-\left(\frac{\left( TMdi- TMcu\right)}{\left( TMci- TMcu\right)}\right)\right]\times 100$

Where *TMdi* = tail moment of drug-treated irradiated sample; *TMcu* = tail moment of untreated, unirradiated control; *TMci* = tail moment of untreated, irradiated control.

### Measurement of SG2000-induced cell cycle alterations using flow cytometry

Exponentially growing cells (2×10^6^cells/mL) were seeded in 6 well plates and incubated at 37 °C in 5 % CO_2_ overnight. Cells were treated with SG2000 (GI_50_ dose) for 1 h and subsequently incubated for up to 72 h in drug free media; untreated cells were used as controls. SG2000-treated cells and corresponding untreated controls were harvested by trypsinisation at specific times post-incubation. Cells were pelleted at 200 g for 15 min at 4 °C, washed with cold phosphate buffered saline (PBS), centrifuged and fixed with ice cold 70 % ethanol added drop-wise (samples could be kept at -20 °C for up to 1 week).

Ethanol was removed from fixed samples by centrifugation at 200 g for 5 min at 4 °C and cells were re-suspended in 400 μL of propidium iodide (PI) staining solution (0.05 mg/ml of PI, 0.5 mg/ml of RNAse A and PBS, to a volume of 5 mL). Samples were incubated for 45 min at 37 °C in the dark and processed on a CyAn ADP High-Performance Flow Cytometer (Beckman Coulter, High Wycombe, UK). Gates were drawn to quantify single cells and eliminate debris and doublets (clumped cells). All reagents were obtained from Sigma-Aldrich Co. unless stated.

Analysis of the red fluorescence from PI nuclear staining was performed using Summit 4.3 software (Dako Colorado Inc. Colorado, USA) to quantify the percentage of cells in each phase of the cell cycle.

### Measurement of γ-H2AX foci *in vitro* by immunofluorescence

For *in vitro* studies, exponentially growing cells (2 × 10^4^ cells/well) were seeded overnight in Nunc™ Lab-Tek™ chamber slides and incubated in a humidified atmosphere at 37 °C and 5 % CO_2_. Cells were treated with SG2000 (0.1, 1, 10, 100 nM) at 37 °C and 5 % CO_2_ for 1 h and then fixed after the appropriate post-incubation time with 1 mL of ice-cold methanol-acetone 50:50 (VWR International, UK) for 8 min at 4 °C. Cells were washed twice with cold PBS and permeabilised (0.5 % Triton-X in PBS) for 15 min at room temperature. Permeabilisation buffer was replaced with 1 mL of cold blocking buffer (0.1 % Triton-X, 0.2 % skimmed dry milk in PBS) at 4 °C overnight. Blocking buffer was washed 3 times with cold PBS and cells were subsequently incubated with 500uL of primary mouse monoclonal antibody anti-phospho-histone H2AX (Ser139) clone JBW301 (Millipore (UK) Ltd, UK) (1:1000 dilution in blocking buffer) overnight at 4 °C. Unbound primary antibody was removed by washing three times for 5 min with washing buffer (0.1 % Triton-X in PBS) and cells were incubated with Alexa Fluor® 488 goat anti-mouse secondary antibody (Life Technologies UK) (1:1000 dilution in blocking buffer) for 4 h at room temperature in the dark. Unbound secondary antibody was removed by washing three times with washing buffer for 5 min and the nuclei were counterstained with PI (2 μg/mL) for 2 min in the dark. Slides were separated from the chambers, rinsed with distilled water for 30 min and allowed to dry in the dark. Slides were mounted with Vectashield (Vector Laboratories, Peterborough, UK), a cover slip (25×60 mm, VWR international, Leicestershire, UK) and the edges of the coverslip sealed with clear nail polish.

Images were visualised with an Axiovert Zeiss Microscope equipped with Perkin Elmer Spinning Disc Technology with two channels (488 nm and 514 nm lasers) and CCD camera (Perkin Elmer, UK) using a 63× oil immersion objective. Nuclear foci were counted in 50 cells per time point and results were expressed as mean number of foci per cell (mean ± SEM) from three independent experiments with Velocity™ Acquisition/Visualisation software (Perkin Elmer, UK).

### *In vivo* studies

All animal experiments were performed in accordance with the UK Home Office Animals Scientific Procedures Act 1986, and United Kingdom Co-ordinating Committee on Cancer Research Guidelines for the Welfare and Use of Animals in Cancer Research [21].

Subcutaneous tumour xenografts were established in CD1 Nu/Nu immunocompromised female mice (Animal Production Facility at the Royal Free Hospital, London) for subsequent therapy and pharmacodynamic (PD) studies. A homogenous suspension of 5 × 10^6^ exponentially growing canine melanoma cells in serum free RPMI 1640 tissue culture medium was injected subcutaneously into the right flank.

Once the xenografts were established, the mice were divided into five groups of ten mice, with equivalent mean tumour volumes for each group. SG2000 was given intravenously into the tail vein to four of these groups and vehicle control was administered to the fifth group. Six mice in each group were used for the study of the anti-tumour effect; tumour and body weight measurements were taken every 3-4 days throughout the study. The remaining four mice in the group were used for the measurement of SG2000-induced pharmacodynamic (PD) endpoints: DNA ICL formation and γ-H2AX foci/cell, in peripheral blood mononuclear cells and tumour tissue. These PD endpoints were measured 2 and 24 h following SG2000 injection. These time points were chosen to capture the peak of ICL and γ-H2AX, respectively, as determined from *in vitro* experiments.

Tumours were measured every 3-4 days with digital calipers until tumour masses reached a maximum size of 1.5 cm^3^. The greatest longitudinal diameter (length), transverse diameter (width) and depth were measured to calculate the tumour volume using the formula for an ellipsoid in cm^3^ [22].

#### SG2000 dosing

SG2000 injections were prepared in 0.9 % NaCl and 1 % DMSO vehicle. Animals were weighed prior to the injection to determine the volume of SG2000 required (0.1 mL/10 g body weight) which was given IV in the tail vein. Control group mice were given an IV injection of the vehicle solution; mice in groups one and two were given a single treatment of SG2000, 0.15 mg/kg and 0.30 mg/kg respectively; mice in groups four and five were given 0.15 mg/kg and 0.30 mg/kg respectively, every seven days for a total of three treatments. These dosing strategies have been used previously with SG2000 [7, 10].

#### Tumour tissue sampling for pharmacodynamics studies

Subcutaneously growing tumour xenografts were dissected with a sharp scalpel blade and kept in cold RPMI 1640 media for immediate processing. Within 30 min, the collected sample was placed in a petri dish over ice. Tumour tissue was finely cut with scalpels, using a cross cutting action, to achieve a single cell suspension. The cell suspension was transferred to a falcon tube containing 5 mL of cold RPMI 1640 media and centrifuged at 200 g for 5 min at 4 °C. The pellet was re-suspended in freezing media (FCS containing 10 % DMSO) and stored at -80 °C.

#### Isolation of peripheral blood mononuclear cells from peripheral blood (Ficoll technique)

Blood was collected from the heart by cardiac puncture or from the tail vein by capillarity. Within 30 min of collection, 100 μL blood was layered carefully onto 500 μL of Ficoll-Paque TM Plus (GE Healthcare Bioscience, UK) in an Eppendorf tube inside a 25 mL universal tube. Blood samples were centrifuged at 450 g for 20 min at room temperature allowing the centrifuge to stop without using the brake. The fluffy mononuclear layer was carefully removed using a Pasteur pipette and the volume made up to 5 mL with cold RPMI 1640 media in a 15 mL Falcon tube. Peripheral blood mononuclear cells were centrifuged at 200 g for 5 min at 4 °C and the pellet was re-suspended in freezing media and stored at -80 °C prior to analysis.

### Measurement of γ-H2AX foci in tumour cells and peripheral blood mononuclear cells by immunofluorescence

Tumour cells and peripheral blood mononuclear cells were fixed in suspension prior to adhesion to the slide. Samples were thawed on ice, re-suspended in 5 mL of cold PBS and centrifuged at 430 g for 5 min at room temperature. Cells were fixed with 2 % paraformaldehyde (PFA) in PBS at room temperature for 20 min. Following centrifugation at 430 g for 5 min, the supernatant was discarded and the cell pellet resuspended in 1 mL PBS. The centrifugation step was repeated as above, the supernatant discarded and the cell pellet re-suspended in 1 mL 70 % ice cold ethanol and incubated on ice for 10 min. The cells were centrifuged at 430 g for 5 min, the supernatant discarded and the cell pellet re-suspended in 200 μL ice cold ethanol. The cells were then transferred to a Shandon EZ single cytofunnel and centrifuged at 47 g with low acceleration for 5 min at room temperature. The slides were removed and allowed to dry at room temperature. Cells were washed twice with cold PBS and permeabilised (0.5 % triton-X in PBS) for 15 min at room temperature followed by blocking (0.1 % triton-X, 0.2 % skimmed milk powder in PBS) overnight at 4 °C. The cells were washed three times with cold PBS and cells were subsequently incubated with mouse monoclonal antibody anti-phospho-histone H2A.X (Ser139) clone JBW301 biotin-conjugated monoclonal primary antibody (Millipore (UK) Ltd,, UK) (dilution 1:1000 in blocking buffer) overnight at 4 °C. Unbound primary antibody was removed by washing three times for 5 min with washing buffer (0.1 % Triton-X in PBS) and cells were incubated with streptavidin conjugated Alexa Fluor® 488 goat anti-mouse secondary antibody (Life Technologies, UK) (1:1000 dilution in blocking buffer) for 4 h at room temperature in the dark. Unbound secondary antibody was removed by washing three times with washing buffer for 5 min and the nuclei were counterstained with PI (2 μg/mL) for 2 min in the dark. Slides were mounted with Vectashield (Vector Laboratories, UK), and the edges of the coverslip sealed with clear nail polish.

### Measurement of γ-H2AX in tumour tissue by immunohistochemistry

Dissected tissue was fixed by complete immersion in a 10 % formalin solution for 24 h at room temperature. Formalin-fixed paraffin wax-embedded tumour xenograft tissues were sectioned with an AO rotary microtome (RM2125 RTS, Leica Microsystems, Milton Keynes, UK). Mounted slides were allowed to dry on a slide warmer at 35 °C for 30 min followed by placing in a dry oven at 100 °C overnight.

Tumour tissue sections on slides were de-waxed in histoclear (National Diagnostics, Hull, UK) for 15 min and dehydrated, first with 100 % IMS (industrial methylated spirits) and subsequently with 70 % IMS/H_2_O, for 15 min. Endogenous peroxidase activity was blocked with 3 % H_2_O_2_/PBS for 20 min. Slides were washed in PBS and antigen retrieval was achieved using 10 mM citrate buffer (3.84 g citric acid in 2 L of H_2_O and addition of sufficient 2 M NaOH to give pH 6) at full microwave power (800 W) for 30 min. Non-specific binding was blocked with 3 % normal goat serum for 20 min at room temperature and incubated with mouse monoclonal antibody anti-phospho-histone H2A.X (Ser139) clone JBW301 (Millipore (UK) Ltd, UK)(dilution 1:200 in 3 % normal goat serum) for 1 h at room temperature. Excess antibody was removed by washing the sections three times each for 5 min with PBS. Anti-antibody **γ**-H2AX bound to the sections was incubated with Vectastain avidin-biotin peroxidase complex (ABC) reagent (Vector Laboratories, UK) for 30 min at room temperature. The sections were subsequently washed three times for 15 min with PBS and peroxidase activity was visualised by the formation of a brown product following addition of labelled polymer-horseradish peroxidase (HRP) diaminobenzidine tetrahydrochloride (DAB) (Sigma, UK) for 5 min. Slides were washed with PBS for 15 min and counterstained with hematoxylin (Harris) (Thermo Scientific) for one minute. Sections were hydrated with 70 % IMS / H_2_O, followed by 100 % IMS, cleared with histoclear for 15 min and mounted with a 50 × 24 mm coverslip and DPX (VWR International).

Sections were visualised using an Axioskop bright field microscope (Carl Zeiss Ltd. Hertfordshire, UK). Images were captured by an AxioCam digital camera and analysed with KS 300 image analysis software (Carl Zeiss Ltd.).

### Statistical analysis

Data were analysed using the GraphPad Prism 6.0 software. Differences in the *in vivo* formation and persistence of ICL in tumour tissue and peripheral blood mononuclear cells following SG2000 treatment was assessed by two-tailed Unpaired Students’ *t*-test. Differences in tumour growth and IHC markers between groups were assessed by a 2-way ANOVA and multiple t-tests. Spearman and Pearson correlations were also used for IHC data. Results were considered as statistically significant at a p-value ≤0.05.

## Results and Discussion

### Activity of SG2000 against canine cell lines *in vitro*

The ability of SG2000 to cause growth inhibition in twelve canine cancer cell lines, representing a range of common tumour types in dogs, and two normal canine cell lines was assessed using the SRB assay (except for the cell line C2 which grows partly in suspension and was therefore determined using the MTT assay). Optimal growth conditions for the 14 cell lines were established initially and are shown in Additional file 1: Table S1. The doubling time of the cells ranged from 20 h (CF33) to 40 h (CMeC-1, CF35.Mg). Drug exposure was either for 1 h or continuous (96 h). GI_50_ values (dose of SG2000 which gives 50 % inhibition of cell growth) are presented in Table 1. Composite growth inhibition curves for all cell lines are shown in Additional file 1: Figure S1.

**Table 1:** Effect of SG2000 on the growth of canine cancer cells and canine normal cells following a 1 h or continuous (96 h) exposure

Cell Line	Cell Type	GI_50_ (nM)^a^	
		1 h	Continuous
C2	Mast cell	0.33 ± 0.84^b^	<0.03^b^
ARCE	Mast cell	>100	4.10 ± 0.87
CMeC-1	Melanoma, cutaneous (primary)	>100	17.33 ± 2.33
CMeC-2	Melanoma, cutaneous (derived from CMeC-1, metastatic in mouse model)	>100	11.0 ± 3.78
12	Melanoma, oral	63.63 ± 45	7.83 ± 1.16
KMeC	Melanoma, oral	4.73 ± 2.22	<0.03
LMeC	Melanoma, oral (metastatic mandibular lymph node)	24.0 ± 3.21	1.60 ± 0.79
DH82	Monocyte/macrophage/histiocytic sarcoma	>100	4.0 ± 2.0
A72	Connective tissue tumour	47.0 ± 3.51	0.83 ± 0.07
DEN	Haemangiosarcoma	45.0 ± 2.5	0.75 ± 0.08
D17	Osteosarcoma	47.5 ± 2.5	0.92 ± 0.57
CF33MG	Mammary gland carcinoma	9.0 ± 3.58	0.99 ± 0.01
CF35MG	Mammary gland (normal)	>100	6.0 ± 0.86
MDCK	Kidney epithelial (normal)	>100	28.33 ± 8.33

^a^GI_50_ values are the dose of drug required to inhibit growth by 50 %. Data shown are the mean ± standard deviation from three independent experiments

^b^Data are from the SRB assay except for cell line C2 which grows partly in suspension and where the GI_50_ value was determined using the MTT assay

SG2000 was cytotoxic across the panel of cell lines. A multi-log differential cytotoxic profile was observed between tumour cell lines with GI_50_ values ranging from 0.33 - >100nM following a 1 h exposure, and <0.03 - 17.33nM following continuous exposure. The canine tumour cell lines therefore showed a similar level of *in vitro* sensitivity to human tumour cell lines, which gave a mean GI_50_ of 7.4 nM in the NCI screen [7]. The two normal cell lines gave GI_50_ values >100nM following a 1 h exposure and the MDCK normal kidney line was the least sensitive cell line following continuous drug exposure. The differential cytotoxicity between tumour cells and normal cells therefore indicated a degree of selectivity for some cancer cells which has potentially important implications clinically.

### SG2000-induced DNA interstrand cross-link formation in canine cell lines

**Fig. 1:**
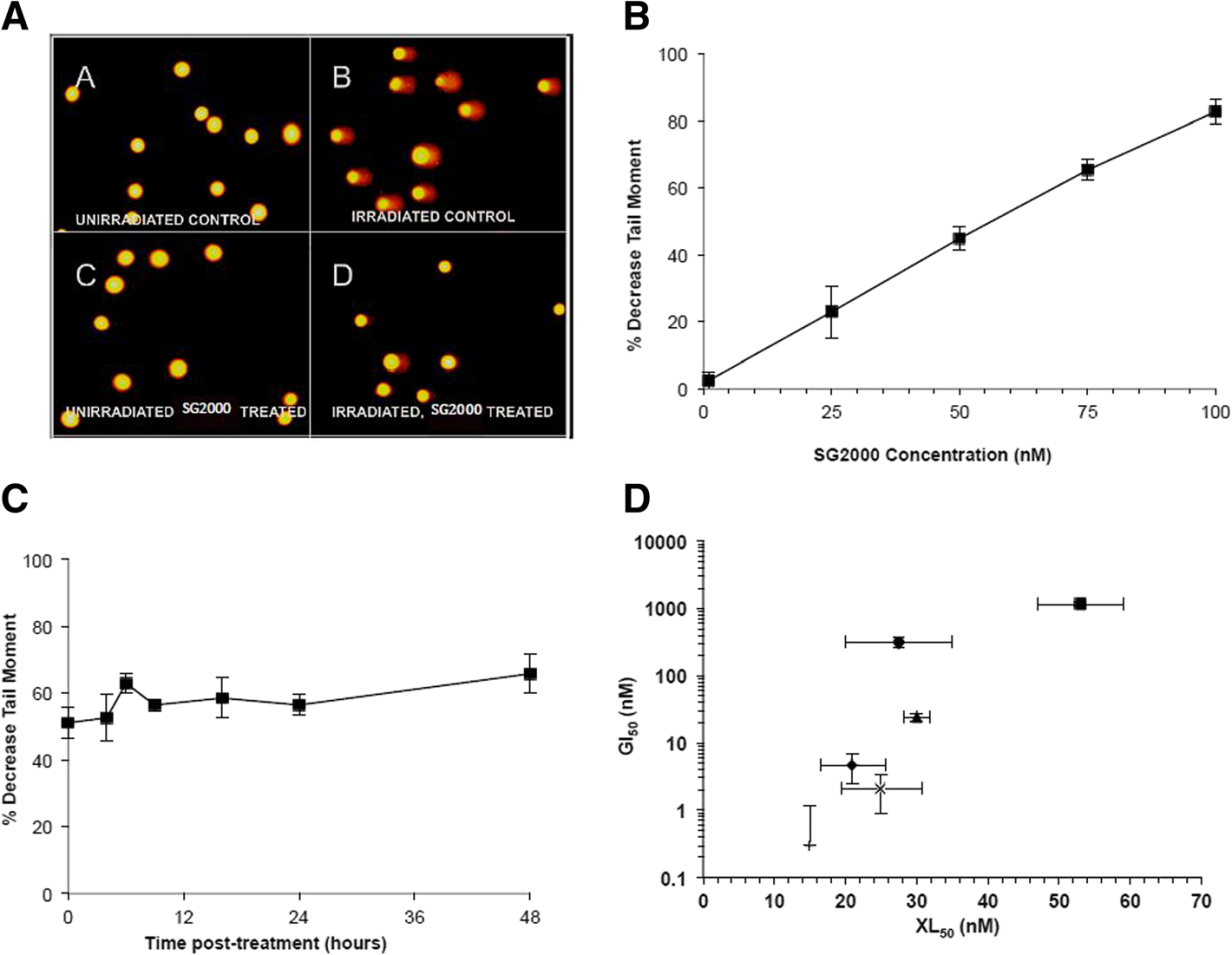
Measurement of DNA interstrand cross-linking using the single cell gel electrophoresis (comet) assay. **a**. Typical comet images obtained from CMeC-1 canine melanoma cells stained with propidium iodide following gel electrophoresis. **a** and **b** are untreated cells and **c** and **d** cells treated with SG2000 for 1 h at 53 nM. Cells were either unirradiated (**a**,**c**) or irradiated with 15 Gy (**b**,**d**) prior to analysis. **b**. Dose-dependent formation of DNA interstrand cross-linking in the CMeC-1 canine melanoma cell line immediately following a 1 h exposure to SG2000. Results are expressed as the mean percentage decrease in the tail moment ± SEM of three independent experiments. **c**. Persistence of SG2000-induced DNA ICLs in the canine melanoma cell line CMeC-1 following a 1 h treatment with SG2000 at 53 nM during 48 h of post-incubation in drug-free medium. Results show the mean percentage decrease of the tail moment ± SEM from three independent experiments. **d**. Relationship between SG2000-induced DNA ICL formation (XL_50_ - dose of SG2000 which gives 50 % decrease in tail moment) versus cytotoxicity (GI_50_ - measured using the SRB assay (Table 1)) of six canine tumour cell lines (C2, ARCE, KMeC, LMeC, CMeC-1 and CMeC-2) and one human melanoma cell line (LOXIMVI). Values represent the mean ± SEM from three independent experiments

DNA ICL formation was studied in canine tumour cell lines following SG2000 treatment using the single cell gel electrophoresis (comet) assay as previously described [19]. Drug treatment was for 1 h and typical comet images are shown in Fig. 1a. It has been shown previously in human tumour cell lines using the same methodology that SG2000-induced DNA ICLs are formed rapidly within 1 h of drug administration [7]. In the CMeC-1 cell line cross-linking (expressed as % decrease in tail moment) was found to increase linearly with dose following a 1 h treatment over a dose range of 1-100nM (Fig. 1b). The persistence of the cross-links over a 48 h period was also examined following a 1 h exposure of CMeC-1 cells to 53nM SG2000, the dose of drug which gives 50 % decrease in tail moment (XL_50_). Fig. 1c shows that the majority of ICL was achieved during the 1 h of drug exposure and that no removal or unhooking of the cross-links is observed over 48 h. This is consistent with previous observations in human tumour cell lines [7].

The relationship between SG2000-induced DNA ICL formation (XL_50_) and cytotoxicity (GI_50_) was examined in six canine cancer cell lines (C2, ARCE, KMeC, LMeC, CMeC-1 and CMeC-2) with differing sensitivities to SG2000 and one human cancer cell line (LOXIMVI melanoma). In Fig. 1d it can be seen that some relationship between ICL formation and cytotoxicity exists (R^2^ = 0.7362). The cell line CMeC-2 is an outlier from the other cell lines and if this cell line is excluded the R^2^ value becomes 0.9131. Although capable of producing other types of DNA damage [8, 9], these data suggest that the DNA ICL produced by SG2000 is a critical cytotoxic lesions.

### Effect of SG2000 on the cell cycle in canine tumour cells

**Fig. 2:**
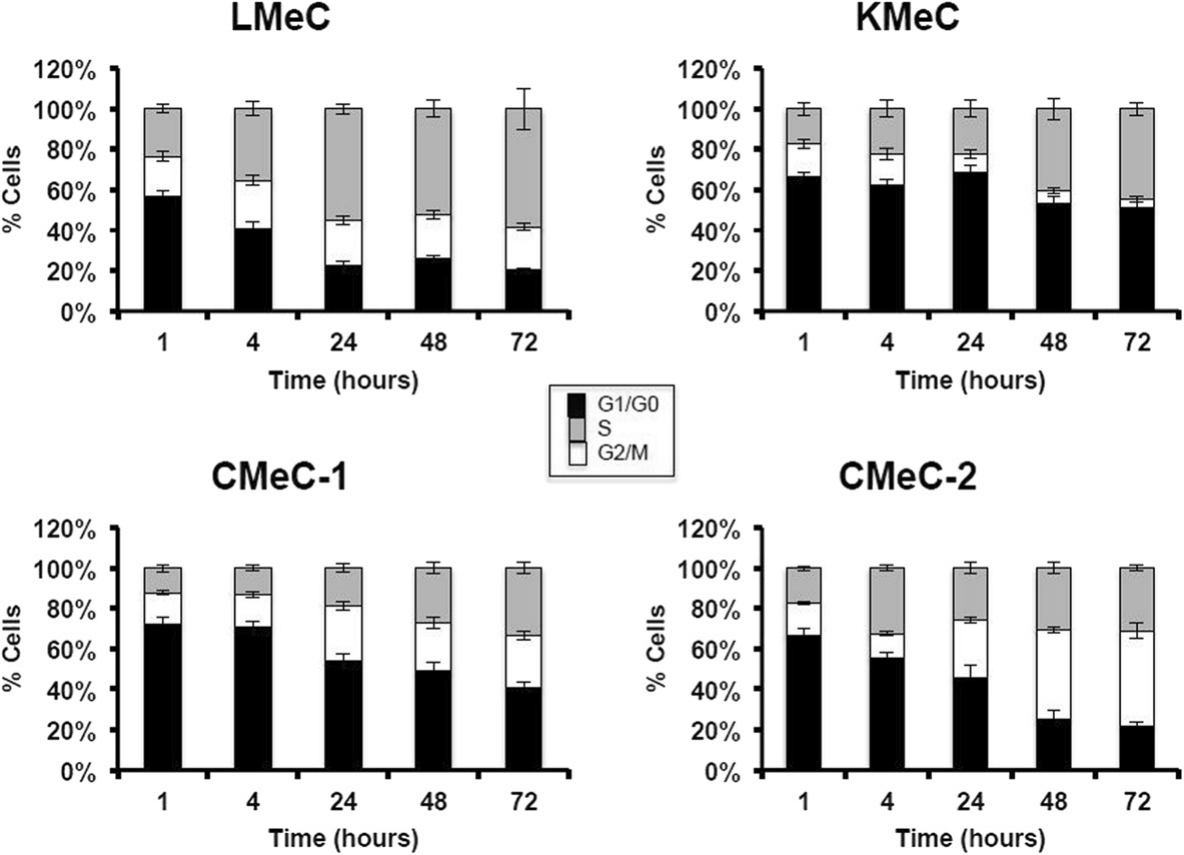
SG2000-induced cell cycle alterations in four canine melanoma cell lines following a 1 h treatment with equitoxic (GI_50_) doses of SG2000 and 72 h of post-incubation. The doses used were LMeC 24nM, KMeC 4.7 nM, CMeC-1 1 μM and CMeC-2 300 nM. Values represent the percentage of cells in the G_1_/G_0_ (black), S (white), and G_2_/M (grey) phases of the cell cycle according to DNA content per cell, assessed by flow cytometry. The percentage of cells in each phase was calculated from a minimum of 10,000 events and the values shown represent the mean from three independent experiments ± SEM

SG2000-induced cell cycle alterations were studied for up to 72 h following a 1 h treatment at equi-toxic (GI_50_) doses in four canine melanoma cell lines (CMeC-1, CMeC-2, KMeC and LMeC). The percentage of cells in G1/G0, S and G2/M are shown at 1, 4, 24, 48 and 72 h in Fig. 2 for the four cell lines. Data exclude sub-G1 (apoptotic) cells, which constituted only a small percentage of cells at the doses used. For example, in CMeC-1 cells, apoptotic cells increased from 2.4 % at time 0 to 8.8 % at 72 h under the conditions employed. An overall decrease in the number of cells in G1/G0 with time was observed in all four cell lines. A clear increase in the number of cells in G2/M was observed in the LMeC, KMeC and CMeC-1 cell lines as has been observed previously in human tumour cell lines [7, 23], indicating that the drug induces cell cycle arrest at this stage of the cell cycle. The exception, however, was cell line CMeC-2 which showed an accumulation of cells in S phase indicating a different cell cycle arrest response in these cells. It is interesting to note that this cell line did not give the same relationship between cytotoxicity and cross-link formation as the other cancer cell lines (Fig. 1d).

### γ-H2AX foci formation following SG2000 treatment of canine cancer cell lines

**Fig. 3:**
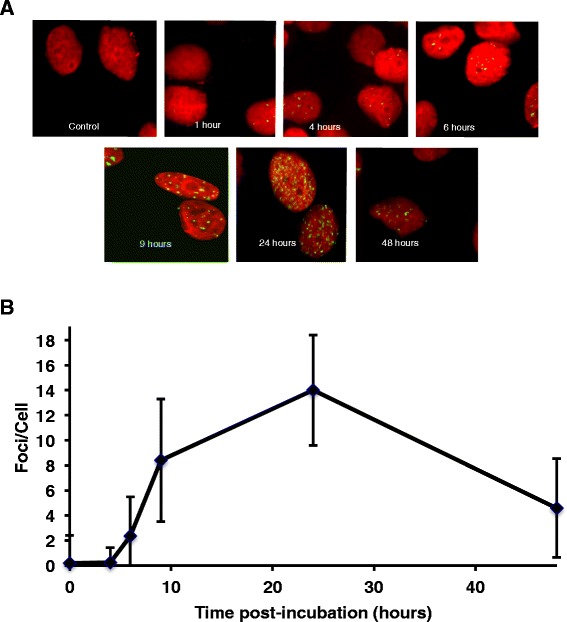
**a**. Immunofluorescence detection of γH2AX phosphorylation in the canine melanoma CMeC-1 cell line following a 1 h treatment with 10 nM SG2000 and up to 48 h of post-incubation in drug-free medium. Immunofluorescence detection of phosphorylated H2AX (γH2AX) appears as green foci. Cellular DNA was counter-stained red with propidium iodide. Representative cells are shown x 63 magnification. **b**. SG2000-induced γH2AX foci/cell in the CMeC-1 canine melanoma cell line following a 1 h treatment with SG2000. Values represent the mean number of γH2AX foci/cell (50 cells per experiment) ± SEM, from three independent experiments. Results were normalised with the mean γH2AX foci/cell of untreated control cells

We have previously shown that exposure of cells to conventional cross-linking agents and SG2000 can induce γ-H2AX foci, clusters of modified histones formed at sites of complex DNA damage [23–25]. In this study the response to SG2000-induced DNA damage was investigated by measuring SG2000-induced γ-H2AX foci in the cutaneous (CMeC-1) and oral (LMeC) canine melanoma cell lines. Cells were treated for 1 h with SG2000 and then incubated for up to a further 48 h in drug-free medium. Typical cell images showing the immunofluorescence detection of γ-H2AX foci in CMeC-1 cells following a 1 h exposure to 10nM SG2000 are shown in Fig. 3a. Foci were scored compared to untreated controls and the time course of formation in CMeC-1 cells is shown in Fig. 3b. Despite cross-links forming rapidly within 1 h (Fig. 1c), the γ-H2AX foci response is slow, only becoming evident after 4 h and reaching a peak at 24 h, after which there is a gradual decline up to 48 h.

The data clearly show that the γ-H2AX assay can detect drug-induced DNA damage response at lower doses than direct measurement of DNA ICL using the comet assay, as we have shown previously [25]. Interestingly, the γ-H2AX foci response to SG2000-induced DNA damage is markedly different to conventional cross-linking drugs. We have previously shown that the peak of γ-H2AX foci formation is approximately 1 h following the peak of ICL (measured using the comet assay) for nitrogen mustard and platinum drugs [24]. In the case of SG2000, however, the current study in canine tumour cells shows that the γ-H2AX response does not peak until 24 h after the peak of the cross-linking. This is consistent with our previous findings in human cells [25] and may reflect the non-distorting nature of the cross-links produced by this agent [5, 7] which may evade early detection by DNA damage response and repair mechanisms.

**Fig. 4:**
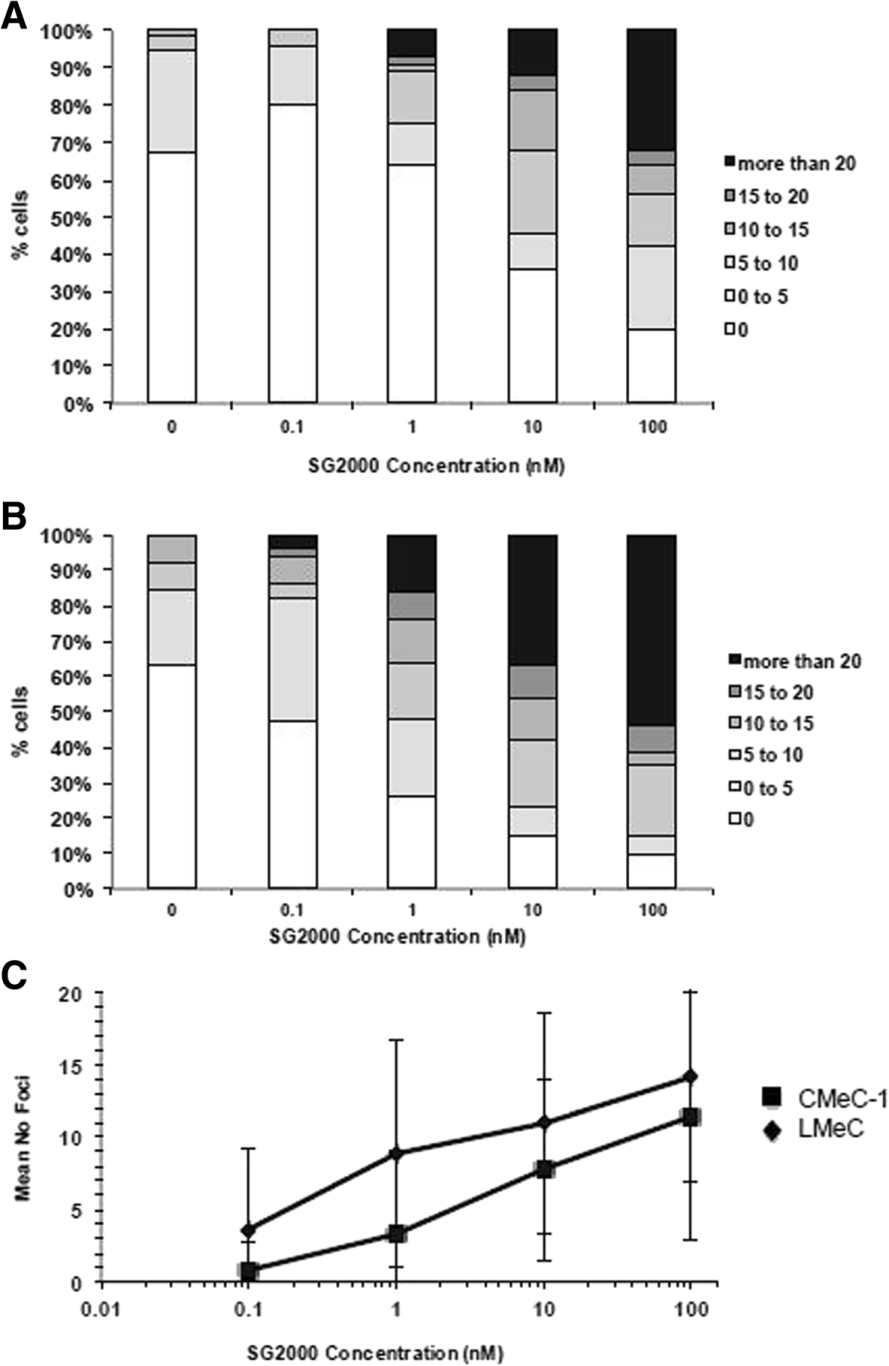
SG2000-induced γH2AX foci/cell in **a**. CMeC-1 and **b**. LMeC cell lines following a 1 h treatment with SG2000 and 24 h post-incubation in drug-free medium. Values shown represent the mean percentage distribution of γH2AX foci/cell (50 cells per experiment) from three independent experiments. **c**. Mean number of foci/cell from CMeC-1 and LMeC cells following treatment for 1 h with SG2000 at the doses shown followed by 24 h post-incubation in drug-free medium. Values are the mean ± SEM from three independent experiments

The effect of dose of SG2000 on γ-H2AX response was determined in both the CMeC-1 and LMeC cell lines (Fig. 4). LMeC cells (Fig. 4b) showed a greater level of background γ-H2AX foci compared to CMeC-1 cells (Fig. 4a). A clear dose response was observed in both cell lines, with greater numbers of foci/cell in LMeC cells at all doses tested (Fig. 4c). This is consistent with the increased sensitivity of this cell line to SG2000. Despite the clear dose-dependent effect, the response was heterogeneous in the cell population at all doses as can be seen by the large errors when the data are expressed as mean foci/cell (Fig. 4c). This can be clearly seen from Fig. 4a and b where in both cell lines at the highest dose of 100 nM there are still >10 % cells which have less than 5 foci per cell. Since γ-H2AX is probably marking sites of DNA double strand breaks generated during lesion processing by structure specific endonucleases, this heterogeneous response may reflect cells in different phases of the cell cycle.

### Efficacy of SG2000 against canine melanoma xenograft tumours *in vivo*

Subcutaneous tumour xenografts in mice were used to investigate whether the observed *in vitro* cytotoxic activity of SG2000 in canine tumour cell lines translated into *in vivo* anti-tumour activity. Melanoma cell lines were chosen for *in vivo* xenograft studies since melanoma is a common canine tumour, constituting 7 % of all malignant neoplasms, and is the most common oral malignancy in dogs, being highly metastatic from this site and resistant to most conventional chemotherapy agents [26]. CMeC-1 and LMeC cell lines were selected, following the preliminary studies on the four canine melanoma lines, due to their ability to establish as subcutaneous tumours in CD1 Nu/Nu immunocompromised mice. CMeC-1 was established from a primary cutaneous melanoma and LMeC was established from a metastatic mandibular lymph node where the primary tumour originated in the oral cavity. Cutaneous and oral melanomas usually have very different biological behaviours in dogs, with the cutaneous form typically being behaviourally benign and the oral form usually being highly malignant and metastatic, therefore we might expect these two cell lines to respond differently to therapy. *In vitro* LMeC was approximately ten-fold more sensitive to SG2000 than CMeC-1 (GI_50_ values 1.6 nM and 17.33 nM after 96 h exposure, respectively).

**Fig. 5:**
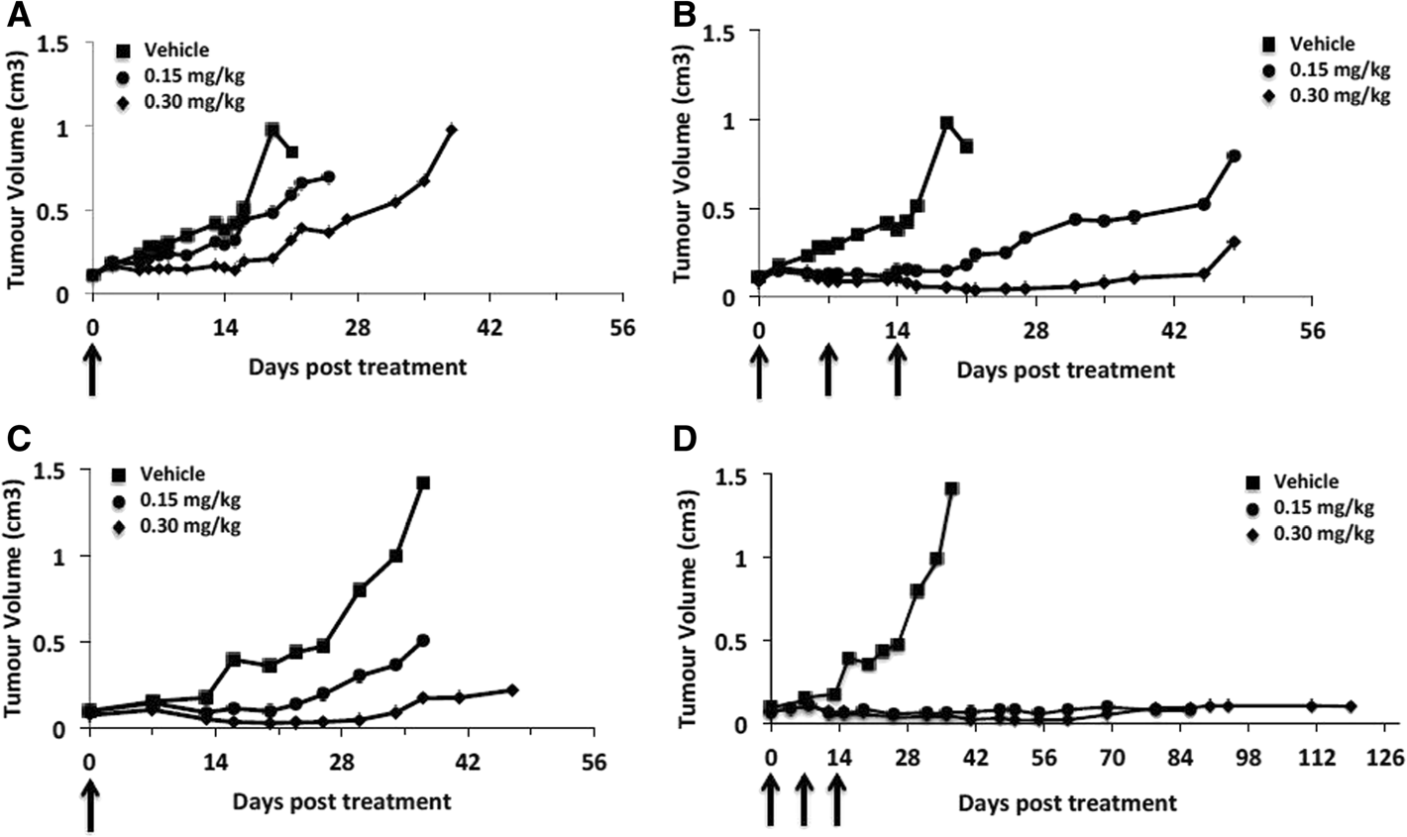
SG2000-induced anti-tumour activity in LMeC (**a**,**b**) or CMeC-1 (**c**,**d**) canine melanoma tumour xenografts in CD1 Nu/Nu nude mice. **a** and **c** are with a single dose of SG2000 i.v. at 0.15 mg/kg or 0.30 mg/kg compared to vehicle only controls. Median tumour volume (cm^3^) from six mice per group is plotted against time in days. **b** and **d** are as in **a** and **c** but SG2000 is given one dose per week for three weeks i.v. at 0.15 mg/kg [●] or 0.30 mg/kg [♦]

Data from *in vivo* efficacy experiments are shown in Fig. 5 against LMeC (A,B) and CMeC-1 (C,D) tumours. SG2000 was administered at two dose levels (0.15 mg/kg and 0.3 mg/kg) using two dosing schedules: single administration (Fig. 5a, c) and once weekly x 3 (Fig. 5b, d). For LMeC, dose dependent inhibition of tumour growth was observed with both dosing schedules, the repeated dose schedule giving a significantly greater growth delay (*p* < 0.05) which was >40 days at the dose of 0.3 mg/kg weekly × 3. Compared to the untreated control group, the anti-tumour effect was significant in all SG2000 dose schedules (*p* < 0.05), apart from the 0.15 mg/kg single dose (*p* = 0.14). Both mean and median survival was increased over the control group under all treatment conditions (Additional file 1: Table S2). Median percentage weight loss was 0 and 2.45 in the single dose 0.15 and 0.3 mg/kg groups, respectively, indicating that the drug was well tolerated under this schedule. Median percentage weight loss of 1.9 and 15.81 was observed in the repeat dose 0.15 and 0.3 mg/kg groups, respectively, suggesting some toxicity at the higher dose level. Weight regain was, however, observed within 14 days following the final dose of SG2000.

The CMeC-1 tumour grew more slowly *in vivo* in control animals compared to LMeC. Significantly greater anti-tumour effect (*p* < 0.01) was observed following the high dose of SG2000 (0.30 mg/Kg) compared to the low dose (0.15 mg/Kg), either as a single dose (Fig. 5c) or administered once a week for three weeks (Fig. 5d). Similarly to what was observed with LMeC, the three-week dose schedule produced a better anti-tumour effect than the single dose schedule (*p* < 0.05). Overall, the anti-tumour effect was greater against the CMeC-1 tumour than the LMeC, with growth delays >100 days at both doses in the repeated dose schedule. An overall increased survival was observed over controls in all schedules except for the single dose 0.15 mg/kg, with the mean survival increased by 90 days over controls in the repeat dose 0.3 mg/kg group (Additional file 1: Table S3). The drug was again well tolerated with no significant weight loss observed in the single dose schedules. In the repeated dose schedules the median percentage weight loss was 4.4 and 12.6 in the repeated dose 0.15 mg/kg and 0.3 mg/kg groups, respectively, again indicating some toxicity at the higher repeated dose.

SG2000 clearly exhibited significant anti-tumour activity against the two canine melanoma tumour models *in vivo*. Greater activity was observed in CMeC-1 than in LMeC, despite the latter being approximately ten times more sensitive to the drug *in vitro*. This difference in activity could relate to differences in growth rate of the tumours *in vivo*, factors such as differences in tumour architecture and vascularisation [27] or different oxygenation of the tumour masses [28], thereby influencing the delivery of SG2000 in the tumour microenvironment. The reduced sensitivity of LMeC relative to CMeC-1 *in vivo* is somewhat disappointing, since it is the highly metastatic oral form of the disease for which effective systemic drug treatment is sought. It is worth noting, however, that CMeC-1 may not behave like a typical cutaneous melanoma of haired skin since it was metastatic to the lymph node in the original patient [16] and may therefore behave more like a subset of cutaneous melanomas that behave aggressively. Nevertheless, SG2000 showed significant activity against both tumour models *in vivo* and the anti-tumour activity in canine patients with spontaneous tumours might be different to those seen in xenograft models.

### Pharmacodynamic measurement of SG2000-induced DNA interstrand cross-linking

**Fig. 6:**
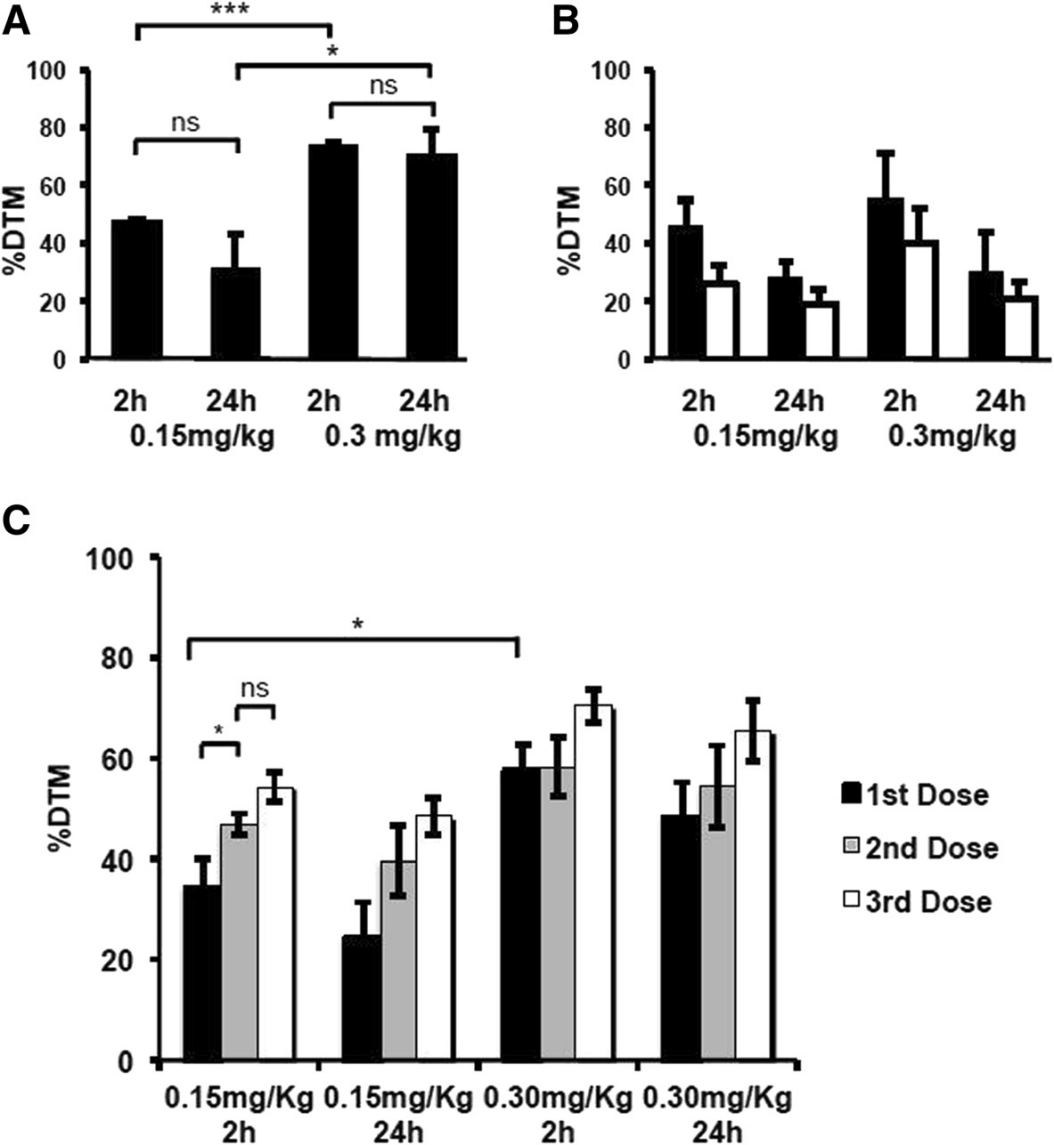
SG2000-induced DNA interstrand cross-linking, expressed as the % decrease in comet tail moment (% DTM) in tumour cells (solid bars) from mice carrying either **a**) LMeC or **b**) CMeC-1 subcutaneous xenografts. Samples were harvested at 2 and 24 h following treatment with either 0.15 mg/kg or 0.30 mg/kg (single dose). Values are the mean % DTM ± SEM calculated from 50 tumour cells each, from three mice each at each time point. In **b**) open bars are the % DTM in peripheral blood mononuclear cells taken at the same time points as the CMeC-1 tumours. **c**. SG2000-induced DNA interstrand cross-linking in peripheral blood mononuclear cells from mice carrying LMeC or CMeC-1 xenografts. Blood samples were harvested from at least three mice at 2 h and from at least three mice at 24 h, following each of three once-weekly doses of SG2000 (either 0.15 mg/kg or 0.30 mg/kg). The % DTM was calculated from 50 cells in each sample as described previously and data are the mean ± SEM. Statistical analysis: *p* ≤ 0.001 = ***, *p* ≤ 0.01 = **, *p* ≤ 0.05 = *, ns = not significant

The formation of ICLs *in vivo* was determined using the single cell gel electrophoresis (comet) assay at doses of SG2000 shown in the above efficacy experiments to give dose-dependent tumour growth inhibition. LMeC tumours were taken after 2 h and 24 h following treatment with 0.15 or 0.3 mg/kg SG2000 and the level of cross-linking determined as the % decrease in tail moment (% DTM, Fig. 6a). A high level of DNA cross-linking was observed in tumour cells at 2 h, consistent with the rapid formation of SG2000-induced cross-links observed in the LMeC cell line *in vitro*. The level of cross-linking in tumour was significantly greater at the higher dose of 0.3 mg/kg, consistent with the increased antitumour effect observed at this dose (Fig. 5). At 24 h cross-links were still evident in tumours at both dose levels (Fig. 6a), consistent with the persistence of the cross-linking observed *in vitro*. A similar experiment was performed in mice bearing CMeC-1 tumours (Fig. 6b), however, additionally in these experiments lymphocytes were also examined for DNA ICL at the same time points as the tumour samples. Dose dependent cross-linking at 2 h and persistence at 24 h was again observed in this tumour *in vivo*. In addition, cross-linking was also observed in lymphocytes, although at a lower level than that observed in tumour cells. This again suggests some degree of selectivity of SG2000 for tumour cells over normal cells.

The measurement of cross-linking described above was following a single administration of SG2000 to mice at 0.15 or 0.3 mg/kg. It is clear from Fig. 5, however, that enhanced antitumour activity is observed against both the LMeC and CMeC-1 melanomas using a weekly repeat dose schedule. The effect of repeated dosing on the level of cross-linking was determined in peripheral blood mononuclear cells since it is possible to take repeated blood samples from the same mouse. It can clearly be seen in Fig. 6c that the level of cross-linking increases following the second and third doses of SG2000 suggesting that a significant level of un-repaired cross-links are still present from the previous drug administration at the time of subsequent dosing. Such persistence of SG2000-induced cross-links was observed in the Phase I study in humans where DNA ICLs correlated with systemic exposure and were still detectable immediately before cycle 2 [14].

### Pharmacodynamic measurement of SG2000-induced γ-H2AX DNA damage response

**Fig. 7:**
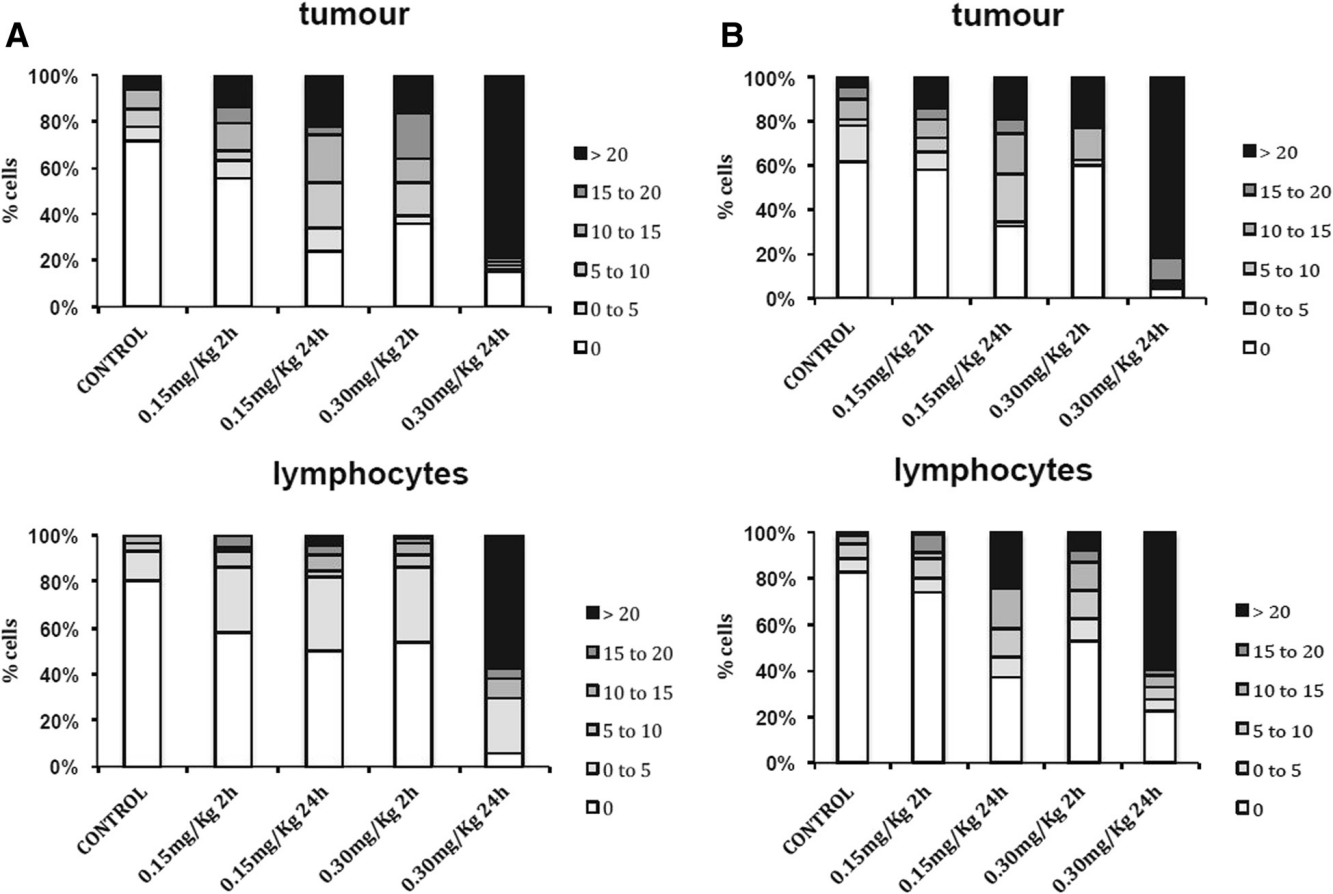
SG2000-induced **γ**-H2AX foci in tumour cells and peripheral blood mononuclear cells (lymphocytes) harvested from three mice carrying subcutaneous **a**) LMeC or **b**) CMeC-1 canine melanoma xenograft tumours, at 2 and 24 h following either 0.15 or 0.3 mg/kg SG2000. γH2AX foci/cell were counted in the first 50 cells and results were grouped in ranges (0, 1–5, 6–10, 11–15, 16–20, and >20 **γ**-H2AX foci/cell)

Despite SG2000-induced cross-links forming rapidly within 1 h, the γ-H2AX foci response *in vitro* is slow, reaching a peak at 24 h (Fig. 3b). This may reflect the non-distorting nature of the cross-links produced by this agent [5, 7] which may evade early detection by the cellular DNA repair machinery. Aliquots of the same samples to those examined for DNA ICL *in vivo* (Fig. 6) were examined for γ-H2AX foci response (Fig. 7). In LMeC tumour cells (Fig. 7a) increased foci were observed compared to untreated tumours at 2 h, with more foci at 0.3 mg/kg compared to 0.15 mg/kg. Consistent with what we observed in canine cells *in vitro* and in previous studies in both human cells *in vitro* and clinical samples [25], at 24 h the number of foci was much greater than at 2 h at both dose levels. A similar dose and time response was observed in CMeC-1 tumours (Fig. 7b).

The γ-H2AX foci response was also determined in peripheral blood mononuclear cells taken at the same time points as the tumours (Fig. 7). The background (control) level of γ-H2AX foci was lower in peripheral blood mononuclear cells than in tumour. Background levels of γ-H2AX have been shown to vary widely between cells in different tissues, culture or different cell lines and it has been found that elevated levels of γ-H2AX are present in a number of human cancer cell model systems [29, 30], suggesting that an increased level of DNA damage is a general characteristic of cancer development. The level of γ-H2AX foci following SG2000 treatment was also greater in tumour than in the corresponding peripheral blood mononuclear cells (Fig. 7). This is consistent with what was observed with DNA ICL in Fig. 6b, again suggesting some degree of selectivity for cancer cells.

### Measurement of SG2000-induced γ-H2AX phosphorylation in tumour tissue using immunohistochemistry

**Fig. 8:**
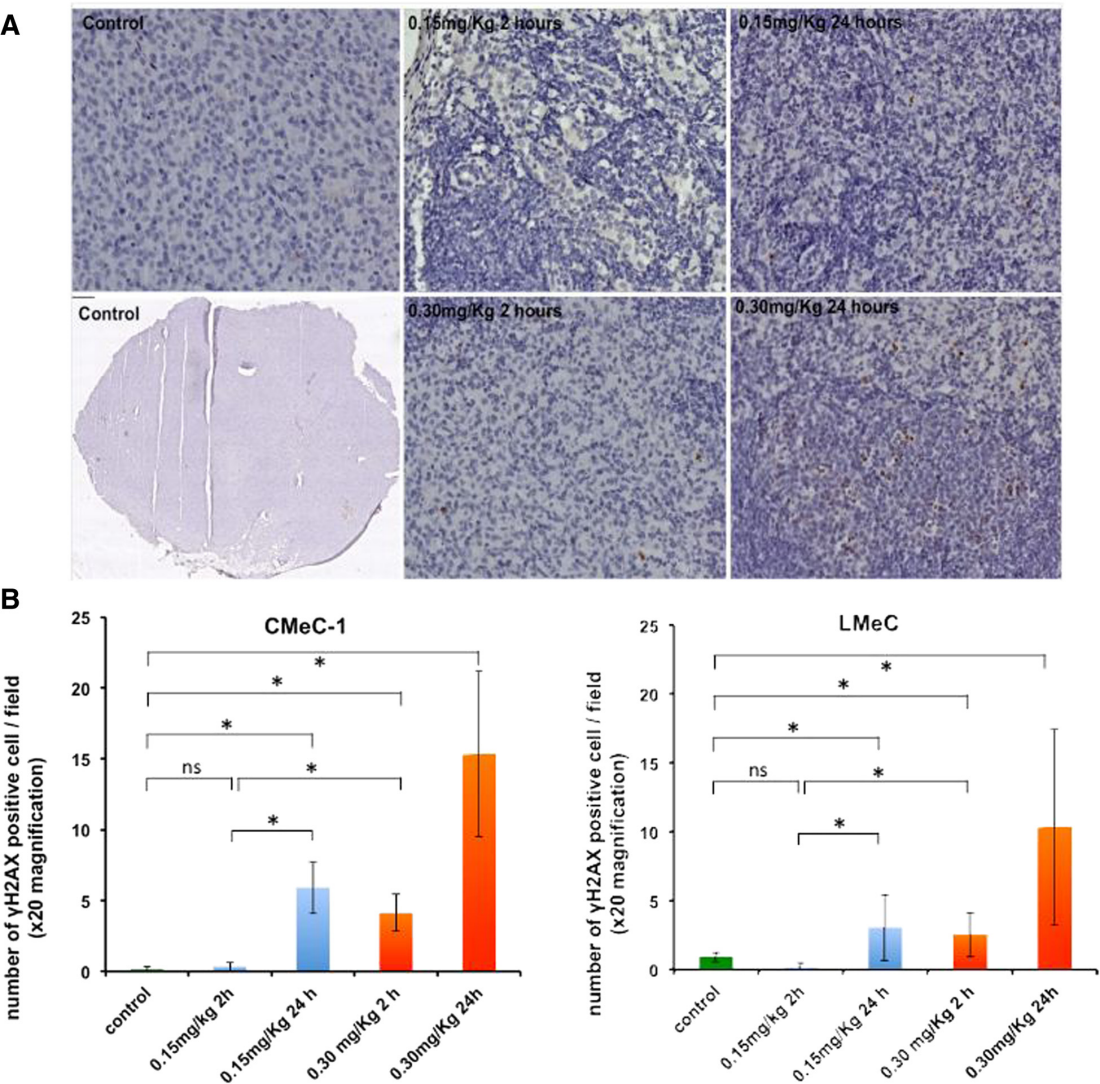
**γ**-H2AX peroxidase staining in paraffin-embedded tissues of canine melanoma xenografts following treatment with SG2000 (single dose of 0.15 mg/Kg or 0.30 mg/Kg). Samples were harvested 2 h and 24 h after administration of the drug. **a**) Examples of fixed tissue sections with **γ**-H2AX peroxidase staining from SG2000-treated CMeC-1 tumour compared to sections from untreated control mice (left). All images are x20 magnification, except the bottom left image that is x10 magnification. **b**) Mean number of **γ**-H2AX positive cells ± SEM per microscopic field (x 20 magnification) in samples from mice bearing either CMeC-1 (left) or LMeC (right) xenograft (sections from three mice were examined in each case). To calculate the mean **γ**-H2AX value, at least 150 cells (at least 50 cells per mouse) and 20 fields per sample were counted. Statistical analysis: *p* ≤ 0.05 = *, ns = not significant

SG2000-induced H2AX phosphorylation was measured in formalin-fixed paraffin-embedded LMeC or CMeC-1 tumour tissue sections. The tumour samples were from the same tumours used for comet and γ-H2AX foci assays. Typical immunohistochemistry images are shown in Fig. 8a, and the number of γ-H2AX positive cells in CMeC-1 and LMeC tumours shown in Fig. 8b.

In both tumour types, a greater number of γ-H2AX positive cells were measured at each time point following 0.30 mg/kg than following 0.15 mg/kg. At each dose, more positive cells were present at 24 h than at two hours after treatment with SG2000, and the effect was greater in CMeC-1 than in LMeC. The number of γ-H2AX positive cells two hours after treatment with 0.15 mg/kg of SG2000 was not significantly different to the number of γ-H2AX positive cells in the untreated control sections. The number of γ-H2AX positive cells 2 h after treatment with 0.3 mg/kg of SG2000 was, however, significantly greater than in the untreated control sections (*p* < 0.05). The number of γ-H2AX positive cells 24 h after treatment with either 0.15 mg/kg or 0.30 mg/kg of SG2000 was significantly greater than the number in the untreated control sections (*p* < 0.05). This was observed in mice carrying either CMeC-1 or LMeC tumour xenografts. These results indicate overall numbers of γ-H2AX positive cells rather than the intensity of staining in a cell, and this assay showed less sensitivity than the γ-H2AX foci technique. However, the data was in good correspondence with that obtained with the latter technique.

## Conclusions

SG2000 demonstrated a similar level of potent *in vitro* activity against a panel of canine tumour cell lines as has been previously demonstrated against human tumour cells. Cytotoxicity correlates with the formation and persistence of sequence selective ICLs in the DNA minor groove as demonstrated using the single cell gel electrophoresis (comet) assay. The cross-links result in a delayed, but robust, γ-H2AX foci response. SG2000 has significant *in vivo* antitumour activity against canine melanoma tumour xenografts, and the comet and γ-H2AX foci methods are shown to be relevant *in vivo* pharmacodynamic assays for measuring DNA ICL and DNA damage response. The clinical testing of SG2000 against canine cancer with associated pharmacodynamic biomarker support is clearly warranted, and a Phase I study in dogs with spontaneous malignancies is currently underway and will be reported in due course.

## Additional file

Additional file 1: Table S1. Specific culture media and supplements required for optimum canine cell lines growth. EMEM - Eagle’s Minimal Essential Medium, DMEM - Dulbecco’s Modified Eagle Medium, RPMI - Royal Park Memorial Institute Medium, FBS – foetal bovine serum, NEAA – non-essential amino acids. **Table S2.** The effect of SG2000 on the survival of LMeC xenografted mice following different SG2000 dosage regimes. **Table S3.** The effect of SG2000 on the survival of CMeC-1 xenografted mice following different SG2000 dosage regimes. **Figure S1.** *In vitro* cytotoxicity of SG2000 against 12 canine tumour cell lines and two canine normal cell lines. Cell viability was assessed using the SRB assay (except for cell line C2 which was assessed using the MTT assay). Results shown are the mean (± SEM) calculated from three independent experiments, expressed as a percentage absorbance at A540 nm compared to untreated control cells. A) 1 h exposure to SG2000 followed by 96 h post-incubation in drug-free medium. B) Continuous (96 h) exposure to SG2000. (PDF 404 kb)
