# Supplementary material for: Activity of the DNA minor groove cross-linking agent SG2000 (SJG-136) against canine tumours
Source: BMC Vet Res. 2015 Aug 19;11:215. doi: 10.1186/s12917-015-0534-2 (PMC4539724; doi:10.1186/s12917-015-0534-2)
Supplement: Additional file 1: Table S1. — Specific culture media and supplements required for optimum canine cell lines growth. EMEM - Eagle’s Minimal Essential Medium, DMEM - Dulbecco’s Modified Eagle Medium, RPMI - Royal Park Memorial Institute Medium, FBS – foetal bovine serum, NEAA – non-essential amino acids. Table S2. The effect of SG2000 on the survival of LMeC xenografted mice following different SG2000 dosage regimes. Table S3. The effect of SG2000 on the survival of CMeC-1 xenografted mice following different SG2000 dosage regimes. Figure S1. In vitro cytotoxicity of SG2000 against 12 canine tumour cell lines and two canine normal cell lines. Cell viability was assessed using the SRB assay (except for cell line C2 which was assessed using the MTT assay). Results shown are the mean (± SEM) calculated from three independent experiments, expressed as a percentage absorbance at A540 nm compared to untreated control cells. A) 1 h exposure to SG2000 followed by 96 h post-incubation in drug-free medium. B) Continuous (96 h) exposure to SG2000. (PDF 404 kb) [file 12917_2015_534_MOESM1_ESM.pdf]

Cell line	Media	FBS	Glutamine	NEAA
**C2**	EMEM	10%	2 mM	1%
**ARCE**	EMEM	5%	10 mM	1%
**CMeC1**	RPMI	10%	2 mM	-
**CMeC2**	RPMI	10%	2 mM	-
**12**	DMEM	10%	2 mM	1%
**KMeC**	RPMI	10%	2 mM	-
**LMeC**	RPMI	10%	2 mM	-
**DH82**	EMEM	10%	2 mM	1%
**A72**	EMEM	5%	2 mM	1%
**DEN**	EMEM	5%+5%New Born FBS	2 mM	-
**D17**	EMEM	10%	2 mM	1%
**CF33**	DMEM	10%	4 mM	-
**CFMg.35**	DMEM	10%	4 mM	-
**MDCK**	EMEM	5%	2 mM	1%

Table S1: Specific culture media and supplements required for optimum canine cell lines growth. EMEM - Eagle's Minimal Essential Medium, DMEM - Dulbecco's Modified Eagle Medium, RPMI - Royal Park Memorial Institute Medium, FBS – foetal bovine serum, NEAA – non-essential amino acids.

Table S2 **The effect of SG2000 on the survival of LMeC xenografted mice following different SG2000 dosage regimes.**

Therapeutic groups	Mean survival ± standard deviation (days)	Median survival (days)	Range (days)
**0.15 mg/kg single dose**	27.16 ± 6.64	27	27 – 38
**0.30 mg/kg single dose**	37.00 ± 10.93	41	22 – 60
**0.15 mg/kg once a week for three weeks**	50.00 ± 8.14	54	48 – 68
**0.30 mg/kg once a week for three weeks**	46.40 ± 12.48	57	35 – 72
**Control**	24.66 ± 8.26	20	21 – 40

Table S3 **The effect of SG2000 on the survival of CMeC-1 xenografted mice following different SG2000 dosage regimes.**

Therapeutic groups	Mean survival ± standard deviation (days)	Median survival (days)	Range (days)
**0.15 mg/kg single dose**	49.16 ± 16.57	41	41 - 81
**0.30 mg/kg single dose**	64.5 ± 24.21	52	41- 99
**0.15 mg/kg once a week for three weeks**	79.20 ± 48.93	72.5	35 - 130
**0.30 mg/kg once a week for three weeks**	130.16 ± 31.37	120	110 - 191
**Control**	41.83 ±8.38	40	30 – 81

A

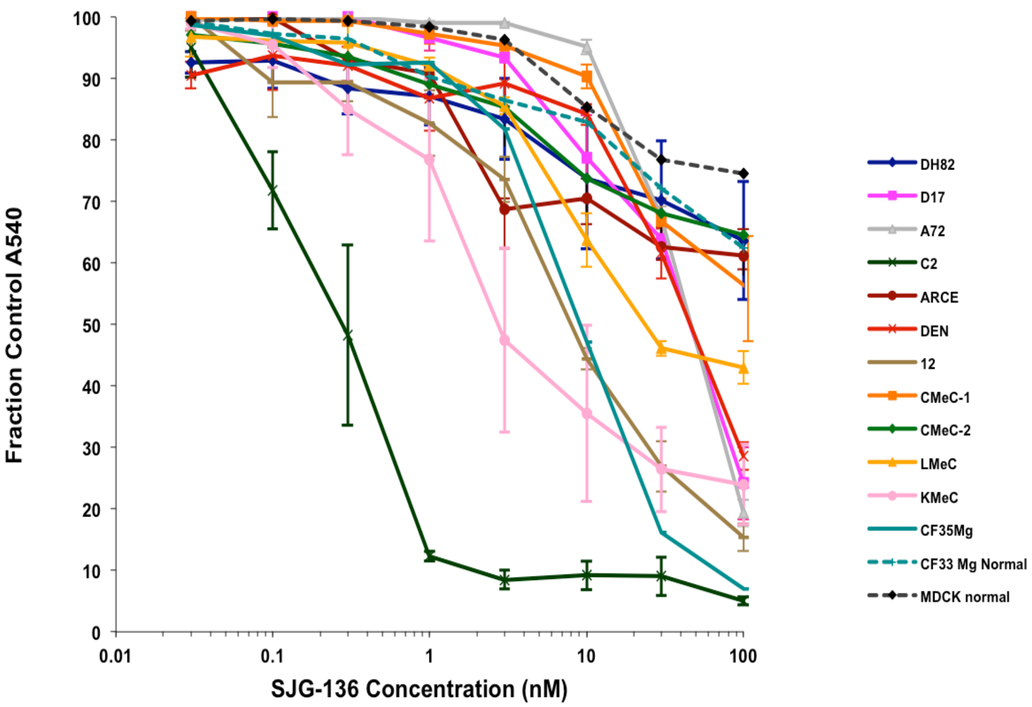

B

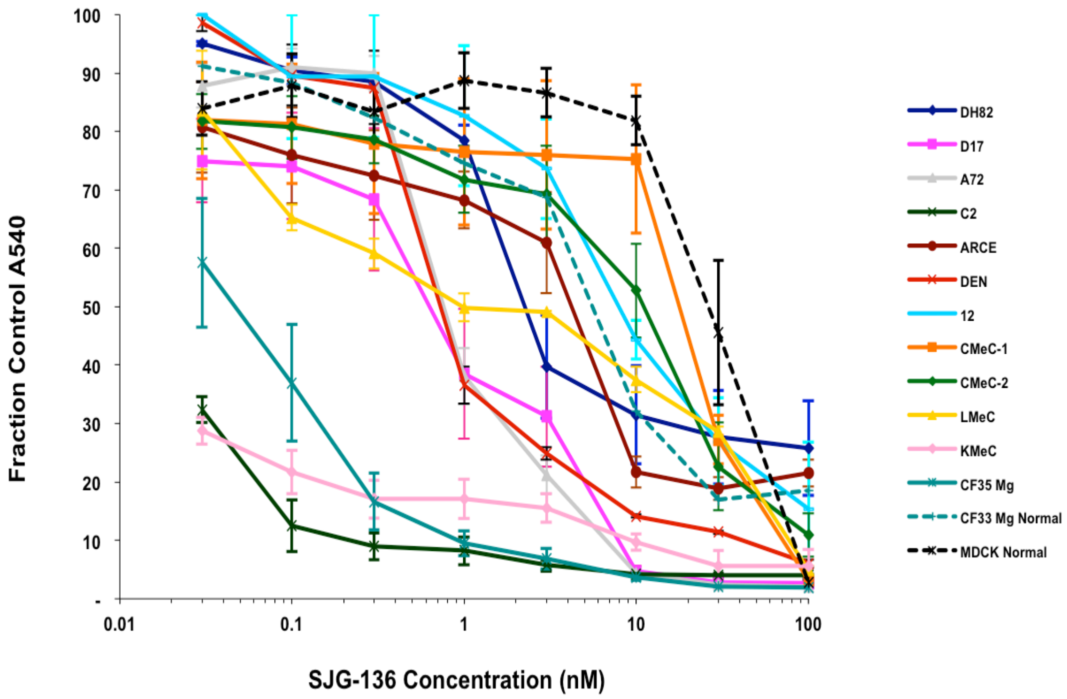

Figure S1 *In vitro* cytotoxicity of SG2000 against 12 canine tumour cell lines and two canine normal cell lines. Cell viability was assessed using the SRB assay (except for cell line C2 which was assessed using the MTT assay). Results shown are the mean (± SEM) calculated from three independent experiments, expressed as a percentage absorbance at A540 compared to untreated control cells. A) 1-hour exposure to SG2000 followed by 96 hours post-incubation in drug-free medium. B) Continuous (96-hour) exposure to SG2000.
